# Visual and Auditory Spatial Localization in Younger and Older Adults

**DOI:** 10.3389/fnagi.2022.838194

**Published:** 2022-04-13

**Authors:** Ying-Zi Xiong, Douglas A. Addleman, Nam Anh Nguyen, Peggy B. Nelson, Gordon E. Legge

**Affiliations:** ^1^Department of Psychology, University of Minnesota, Minneapolis, MN, United States; ^2^Center for Applied and Translational Sensory Science, University of Minnesota, Minneapolis, MN, United States; ^3^Department of Psychological and Brain Sciences, Dartmouth College, Hanover, NH, United States; ^4^Department of Speech-Language-Hearing Sciences, University of Minnesota, Minneapolis, MN, United States

**Keywords:** aging, spatial localization, visual perception, auditory perception, sensory integration

## Abstract

Visual and auditory localization abilities are crucial in real-life tasks such as navigation and social interaction. Aging is frequently accompanied by vision and hearing loss, affecting spatial localization. The purpose of the current study is to elucidate the effect of typical aging on spatial localization and to establish a baseline for older individuals with pathological sensory impairment. Using a verbal report paradigm, we investigated how typical aging affects visual and auditory localization performance, the reliance on vision during sound localization, and sensory integration strategies when localizing audiovisual targets. Fifteen younger adults (*N* = 15, mean age = 26 years) and thirteen older adults (*N* = 13, mean age = 68 years) participated in this study, all with age-adjusted normal vision and hearing based on clinical standards. There were significant localization differences between younger and older adults, with the older group missing peripheral visual stimuli at significantly higher rates, localizing central stimuli as more peripheral, and being less precise in localizing sounds from central locations when compared to younger subjects. Both groups localized auditory targets better when the test space was visible compared to auditory localization when blindfolded. The two groups also exhibited similar patterns of audiovisual integration, showing optimal integration in central locations that was consistent with a Maximum-Likelihood Estimation model, but non-optimal integration in peripheral locations. These findings suggest that, despite the age-related changes in auditory and visual localization, the interactions between vision and hearing are largely preserved in older individuals without pathological sensory impairments.

## Introduction

Robust spatial localization aids real-world behaviors like navigation and social interaction. Vision and hearing are particularly critical for localizing stimuli beyond one’s reach (Long and Giudice, 2010). It’s well-known that age-related vision and hearing loss may impair spatial perception (Subramanian and Dickinson, 2006; Akeroyd, 2014). For the large and increasing aging population with concurrent vision and hearing impairment (Fuller et al., 2018), spatial localization is expected to be even more challenging, which further raises the significance of sensory interactions (Saunders and Echt, 2007; Simon and Levitt, 2007). However, it is difficult to isolate the impacts of pathological sensory impairment on spatial localization from the performance decline due to normal aging. The current study investigates how typical aging affects the spatial localization of visual and auditory stimuli, and the combination of information from the two senses in the localization of audiovisual stimuli. In addition, this study aims to provide a baseline for interpreting our ongoing investigations of the impacts of single and dual sensory impairment on spatial localization.

Aging is associated with changes in visual and auditory pathways from eyes and ears to cortical areas (Andersen, 2012; Recanzone, 2018). Regarding auditory localization, older adults reportedly show reduced accuracy and precision even with age-adjusted normal hearing thresholds (Dobreva et al., 2011, 2012; Freigang et al., 2014, 2015), possibly due to age-related deficits in processing interaural temporal difference, interaural level difference, and monaural spectral cues that are crucial for sound localization (for a review, see Gallun, 2021). Older individuals also show impaired visual spatial abilities despite normal vision, such as reduced accuracy and precision in spatial updating (Bennett et al., 2017) and impaired scene perception which requires integration of global spatial information (Meng et al., 2019). In both visual and auditory localization, the magnitude of age-related deficits varies across spatial locations, with sound localization showing the greatest impairment at peripheral locations (Dobreva et al., 2012), and the visual useful field of view shrinking with increasing age (Sekuler and Ball, 1986; Sekuler et al., 2000). These age-related deficits despite the absence of pathological sensory impairment point to the importance of elucidating the effect of normal aging as a baseline for older people with sensory impairment.

Although vision and hearing are initially coded by different pathways and have different levels of spatial accuracy and precision, the spatial information from the two sensory modalities must be unified to form a coherent multimodal space (King, 2009; Loomis et al., 2012; Postma and Van Der Ham, 2017). The interactions between vision and hearing become increasingly important for people with sensory impairment. In the current study, we focus on three aspects of audiovisual interactions that are important for daily functions.

It is well established that younger individuals have more accurate and precise visual than auditory localization performance, due to the advantage of retinotopic mapping for visual space over the interaural comparison for auditory space (Battaglia et al., 2003; King, 2009). However, it is currently unclear whether the unequal spatial reliabilities of audition and vision change in later life, especially at peripheral locations where aging has the most impact on both visual and auditory localization. Indeed, aging increases the speed at which people respond to visual versus auditory stimuli (Murray et al., 2018), raising the possibility that the relationship between auditory and visual spatial perception also changes with age.

Another important aspect of audiovisual interaction is that the visual environment can provide a reference for aligning visual and auditory information (King, 2009; Long and Giudice, 2010). In younger adults with normal vision and hearing, auditory localization is less precise without vision (e.g., when blindfolded, Tabry et al., 2013; Voss, 2016). However, it is not known if this pattern is also present for older individuals. It is possible that older individuals will show larger benefits from having visual context, especially if they show deficits in sound localization performance despite normal hearing thresholds. Alternatively, the visual input might distract the sound localization, as older individuals have been reported to show larger difficulty in focusing attention to a single modality in the presence of other sensory inputs (Andres et al., 2006).

Finally, audiovisual interaction has been extensively studied in the context of audiovisual integration. Behavioral, brain imaging, and computational approaches have reported that people often integrate modalities in a statistically optimal manner, assigning weights to each modality based on the relative reliability of representations for each modality (e.g., Ernst and Banks, 2002; Kersten and Yuille, 2003). Older individuals reportedly have stronger sensory integration in some tasks, including larger temporal integration windows of visual and auditory targets (Diederich et al., 2008; Murray and Wallace, 2011) and more audiovisual integration in the McGurk effect (Cienkowski and Carney, 2002). The enhanced sensory integration is possibly due to a combination of reduced unisensory reliability, increased tendency to bind information, and changes in response strategies (Baum and Stevenson, 2017; Jones and Noppeney, 2021). It should be noted that stronger sensory integration does not necessarily indicate more optimal integration, for example, the larger temporal integration window can actually reduce the probability of stimulus overlap (Diederich et al., 2008). Despite substantial literature on the effect of age on audiovisual temporal integration, studies on audiovisual integration in the space domain are sparse. It is not clear whether age affects the optimality of integration of visual and auditory spatial information, especially at peripheral locations where both forms of unimodal localization may show large deficits.

The current study aims to investigate the impact of age on visual localization, auditory localization, and the interactions between vision and hearing in localization. We asked younger and older subjects to localize visual, auditory, and audiovisual targets presented in the horizontal plane ranging from straight ahead (0°) to 90° to the left and right. Our design incorporates features that will make the paradigm suitable for future studies with older subjects with sensory impairment. We chose to have subjects provide verbal responses instead of pointing or walking, because verbal report can be easier for older individuals with mobility constraints, and there are advantages over pointing in accuracy (Philbeck et al., 2008). We presented the visual, auditory, and audiovisual stimuli across a 180° range on the horizontal plane instead of a restricted central range as in past literature. We compared auditory localization performance with and without blindfolds to examine the effect of visual environmental context and used modeling to examine the optimality of audiovisual spatial integration. We conclude by commenting on the feasibility of applying our approach to investigate spatial localization in subjects with sensory impairment.

## Materials and Methods

### Subjects

Fifteen younger subjects (eight women and seven men, aged 19–33 years, mean = 26 years) were recruited from the Research Experience Program in the University of Minnesota. Thirteen older subjects (nine women and four men, aged 59–78 years, mean = 68 years) were recruited from the University Retirees Volunteer Center at the University of Minnesota. All subjects self-reported no known vision or hearing disorders. This study was approved by the University of Minnesota Institutional Review Board and followed the Declaration of Helsinki. Consent forms were acquired from all subjects prior to their participation in this study.

For all subjects, normal visual acuity and contrast sensitivity were confirmed by the Lighthouse Distance Visual Acuity chart (Ferris et al., 1982) and Pelli-Robson Contrast Sensitivity chart Low-Vision Version (Pelli et al., 1988), respectively. Subjects completed acuity and contrast sensitivity tests with their most up-to-date prescriptions, if any. All subjects had normal visual acuity (<0.2 logMAR), and there was no significant group difference [Younger: −0.09 ± 0.08 logMAR; Older: −0.04 ± 0.09 logMAR, *t*(26) = 1.56, *p* = 0.13]. All subjects were able to complete the last line on the Pelli-Robson Contrast Sensitivity chart, which corresponds to a contrast sensitivity of 1.65 log unit.

**FIGURE 1:**
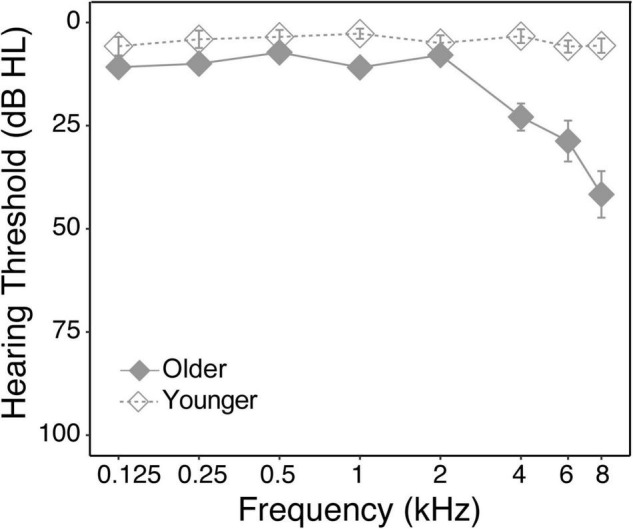
Average pure-tone audiograms for each group. Hearing thresholds (dB HL) were plotted as function of pure-tone frequencies. Solid and empty diamonds represent older and younger groups, respectively.

Hearing thresholds were tested at 125, 250, 500, 1 k, 2 k, 4 k, 6 k, and 8 kHz by pure-tone audiometry using an air conduction audiometer. Figure 1 shows the average hearing thresholds for each group at each tested frequency. Younger subjects had normal hearing thresholds at all frequencies. Older subjects had typical performance for their age (International Organization for Standardization [ISO], 2000), showing normal thresholds (<=20 dB) at low frequencies (125–2 kHz) but mild hearing loss at high frequencies (4 k–8 kHz). Pure-tone average was calculated across 0.5–4 kHz frequencies according to WHO standards. All subjects met the criteria for normal hearing (pure-tone average <=20 dB), with the younger group showing significantly better thresholds [Younger: 0.5 ± 3.5 dB; Older: 11.7 ± 4.3 dB, *t*(26) = 7.2, *p* < 0.001]. All subject had symmetric hearing thresholds (<20 dB difference).

Normal cognitive status was verified by the Mini-Mental State Examination (score >24).

### Apparatus and Stimuli

The testing was conducted in the Multisensory Perception Lab (MSP) in a sound-attenuating chamber with interior dimensions of 10’ × 13’ × 8.5’ (width, length, height). The auditory stimuli were presented using 17 A’Diva ti speakers (Anthony Gallo Acoustics). All of the speakers faced the center of the room, where subjects sat. Visual stimuli were presented by a video projection system ranging 220° in the horizontal plane. The resolution of the video system is 3840 × 720, with a 60-Hz refresh rate. The projector screens are acoustically transparent over a 20-Hz to 20-kHz frequency range. All speakers were occluded behind the projector screens and were not visible to the subjects throughout the experiment. The project fans had an average noise level at 33 dB sound pressure level (SPL), with the noise level decreasing at high frequency (e.g., 13 dB at 8 kHz).

Psychtoolbox 3.0 software (Pelli, 1997) with MATLAB R2016a was used to generate stimuli and control the experimental procedures. Auditory stimuli were pink noise bursts with a frequency band of 200–8,000 Hz. Broadband pink noise was used because it has equal energy per octave, which represents the energy distribution of human hearing, and is also representative of the broadband sounds in daily environments. The stimuli lasted 200 ms (with 50 ms onset/offset ramps), with an intensity of 60 dB SPL. Visual stimuli were bright disks with a diameter of 3°, a duration of 200 ms, and a contrast of 90%.

### Procedure

In the spatial localization experiment, subjects were seated in the center of the booth approximately 1.5 m from the speakers and projectors. There were no additional lighting sources except the projectors. Subjects were asked to always keep their head and gaze straight ahead. Subjects were instructed to localize stimuli presented randomly at one of seventeen locations in steps of 10°, ranging from −90° to 90° azimuth. The −40° and 40° locations were omitted because these locations were at the intersection between the screens where visual stimuli can’t be presented properly. There were four conditions: (1) vision-only, in which only the bright disk was presented; (2) audition-only, in which subjects wore a blindfold while only the noise burst was presented; (3) audition no-blindfold, in which subjects localized the noise burst without wearing a blindfold. In this condition, the projector screen had a dark background without any visual stimuli. However, the layout of the room and the screen-floor boundaries were visible under the dim light of the projectors; and (4) audiovisual condition, where the visual and auditory stimuli were both presented simultaneously from the same location.

**FIGURE 2:**
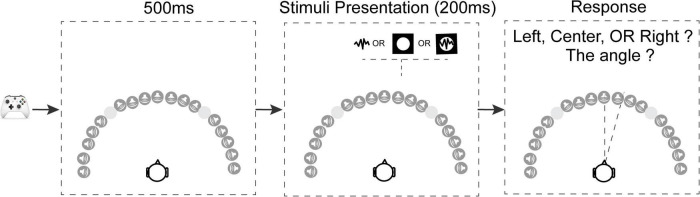
Schematic of the trial structure. Subjects used an Xbox controller to initiate each trial. After the key press there was a 500 ms delay, followed by a stimulus presented from one of the seventeen locations. There were four stimulus conditions: in the vision-only condition a white disk was presented for 200 ms; in two audition conditions (with or without blindfold) pink noise was presented for 200 ms; in the audiovisual condition the white disk and pink noise were presented simultaneously from the same location for 200 ms. After the target presentation, the subjects needed to verbally report the location of the stimuli, first responding left or right, then responding with an angle relative to directly in front of the subject. The –40° and 40° locations were omitted because these locations were at the intersection between the screens where visual stimuli can’t be presented properly.

The procedure for a trial is shown in Figure 2. Subjects pressed a button on an Xbox controller to initiate each trial. After the subject initiated a trial, there was a 500 ms blank interval before stimulus presentation. In each trial, the pink noise, bright disk, or both, were presented randomly at one of the seventeen locations ranging from −90° to 90° azimuth. The subjects’ task was to verbally report the direction that the stimulus originated from, first by reporting its orientation (left, right, or center), then by estimating its location angle (0°–90°) if the stimuli originated from the left or right. Subjects were instructed to be as precise as possible with the angle estimation, using increments of up to 5°. They were encouraged to make their best guess on each trial, but if the subject couldn’t see or hear the stimuli, they were instead allowed to report the trial as a “miss.” Subjects were allowed to take as long as needed to respond. Subjects communicated with the researcher outside the MSP Booth through a two-way sound monitor, and the subjects’ verbal responses were recorded by the researcher.

Before the actual testing began, subjects were given a practice run that included five trials for each condition, which familiarized them with the general procedure and each of the testing conditions. In the actual testing, each of the seventeen directions was tested two times per condition per block. Conditions varied across block with two blocks per condition, resulting in eight blocks of 34 trials per block, or 272 trials per subject. The order of the conditions was randomized for each subject, and subjects were notified of the condition for each block before it started. Subjects were encouraged to take breaks whenever necessary. After every four blocks, subjects were asked to take a mandatory break of at least 30 s. To avoid learning effects, no feedback was given in either practice or testing. The total duration of the experiment was approximately 90 min.

### Data Analysis

Analyses were performed using R software (R Core Team, 2005). Three key localization parameters were obtained for each subject in each condition. Absolute error was calculated as the absolute deviation between the reported location and actual location, representing the magnitude of the response errors. Bias was calculated as the mean signed deviation between the reported and actual location (bias = reported locations – actual locations), representing both the magnitude and direction of any response errors. Overshooting errors refer to reporting stimuli as being more peripheral than their actual location. In the left field negative biases indicate overshooting (e.g., reporting a −10° target as −20°), while in the right field positive bias indicates overshooting (e.g., reporting a 10° target as 20°). Undershooting errors refer to reporting peripheral stimuli as being more central than their actual locations (e.g., reporting a −20° target as −10° in the left field, or reporting a 20° target as 10° in the right field). Variability was calculated as the standard deviation of the responses across the four trials at each location for each condition, with smaller variability representing higher precision.

We constructed linear mixed effect models (LME; Pinheiro and Bates, 2000) on the localization parameters with condition, target location, and group as fixed factors, and subject as a random factor. For all LME models, significant main effects of the fixed factors were examined by the ANOVA function. *Post hoc* analyses were conducted with Bonferroni correction (“emmeans” package, Piepho, 2004).

## Results

### Miss Rate

**FIGURE 3:**
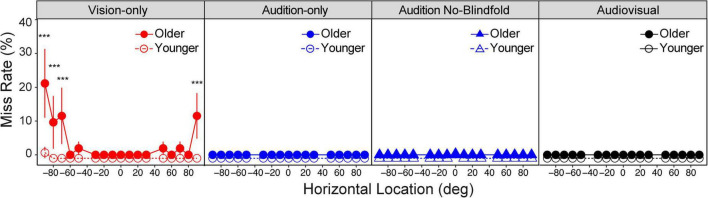
Miss rate. Miss rate in the four conditions for the older and younger groups, respectively. Note that most of the dots overlapped at 0 miss rate, therefore the data for the two groups were slightly offset vertically to make them more distinguishable. The error bars represent standard errors. Statistical asterisks represent significant difference between the older and younger groups. ***p < 0.001.

We first asked whether the two groups reported missing the target at different rates. The miss rate was obtained as the percentage of trials at each location in each condition that the subjects reported “miss.” Overall miss rates were low in both groups, including miss rates near 0% in both groups for the audition-only, audition no-blindfold, and audiovisual conditions (Figure 3). LME model with the miss rate as dependent variable and group, condition and location as fixed factors showed significant main effect of group [*F*(1,26) = 6.43, *p* = 0.018], condition [*F*(3,1742) = 14.80, *p* < 0.001], and location [*F*(16,1742) = 2.81, *p* < 0.001]. There were also significant interactions between each pair of factors (all *ps* < 0.001) and a three-way interaction [*F*(48,1742) = 2.34, *p* < 0.001]. *Post hoc* analysis showed that only the vision-only condition had a significant group difference, with the older group missing significantly more often (3.5% of trials) than the young group (<0.5% of trials) (*p* < 0.001). Further examination showed that older subjects were more likely to miss visual targets in far peripheral locations than younger subjects (see Figure 3). For example, at left 90° the missing rate in the older group reached 22% on average (*p* < 0.001).

### Absolute Error

**FIGURE 4:**
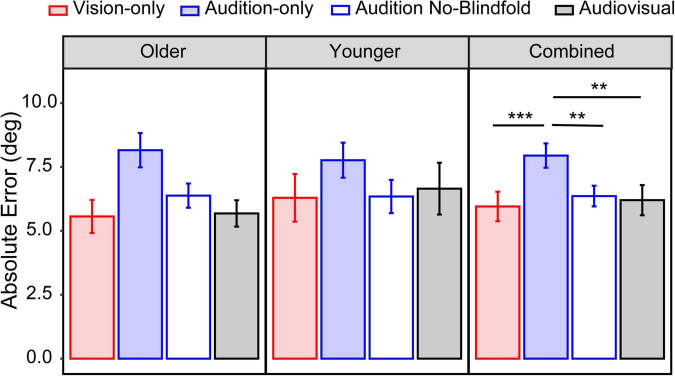
Overall absolute error. The mean absolute error across all seventeen locations in the four conditions for the older group **(left panel)**, younger group **(middle panel)**, and both groups combined **(right panel)**. The error bars represent standard errors. Statistical asterisks represent significant difference between testing conditions. ***p < 0.001; **p < 0.05.

We considered the average absolute error across all seventeen locations as an indicator of the overall accuracy. For the older group, the mean absolute error ranged from 5.6° in the vision-only condition to 8.2° in the audition-only condition (Figure 4, left panel). For the younger group, the mean absolute error ranged from 6.3° in the vision-only condition to 7.8° in the audition-only condition (Figure 4, middle panel). LME modeling showed a significant main effect of condition [*F*(3,78) = 7.84, *p* < 0.001], but not a main effect of group [*F*(1,26) = 0.13, *p* = 0.72] nor an interaction between condition and group [*F*(3,78) = 0.94, *p* = 0.42]. *Post hoc* analyses were thus conducted on the two groups jointly (Figure 4, right panel). The absolute error in the vision-only condition was significantly smaller than the audition-only condition (*p* < 0.001). Interestingly, the audition-no blindfold condition also had significantly smaller absolute error than the audition-only condition (*p* = 0.006), suggesting a benefit of visual context on auditory localization. This visual contextual benefit averaged 1.8° in the older group and 1.4° in the younger group. The absolute error in the audiovisual condition was smaller than the audition-only condition (*p* = 0.001) but similar to the vision-only condition (*p* = 1.00), indicating that the addition of auditory input had little contribution when localizing audiovisual targets.

### Bias

**FIGURE 5:**
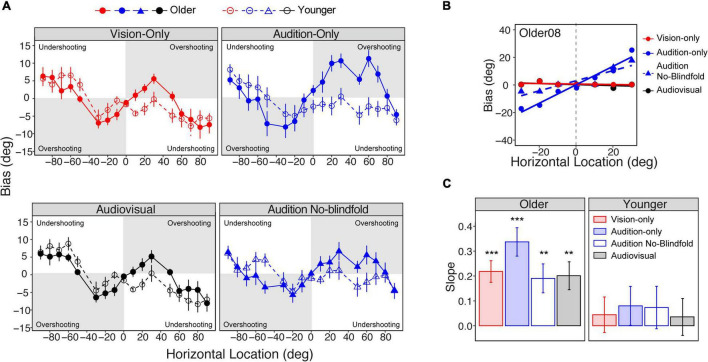
Localization bias. **(A)** The average biases across the seventeen locations in the four conditions for the older and younger groups, respectively. Overshooting and undershooting are annotated on each plot. **(B)** Linear regressions of bias on the central locations (–30° to 30°) in an example subject. **(C)** Bar plots representing the average slopes of the linear regression in the four conditions for the older (left panel) and younger (right panel) groups, respectively. The error bars represent standard errors. Statistical asterisks represent that the slope is significantly larger than 0. ***p < 0.001; **p < 0.05.

Although the two groups showed similar absolute errors, group differences appeared in the pattern of bias across the seventeen locations. Bias was calculated as the difference between the reported locations and actual target locations. As shown in Figure 5A, both groups reported peripheral stimuli as being more central (e.g., report a 90° target as 85°) than their actual locations (undershooting). For stimuli that were more central, the older group tended to report the target as more peripheral (overshooting). The largest overshooting in the older group was observed at ±30° target locations in all modalities, where on average the subjects reported the target to be at ±40°. However, this trend was not shown in the younger group. To quantify this overshooting effect, we fit linear regressions on biases as a function of location for the subset of data from −30° to 30° where the overshooting was most prominent (see Figure 5B for an example). We used the slope of the linear regression as an index of the magnitude of the bias (Dobreva et al., 2011). A positive slope represents overshooting, and a negative slope represents undershooting. Figure 5C shows the mean slopes for the older and younger groups in the four conditions. The older group showed significant overshooting in all four conditions (slopes >0, *ps* < 0.05), with the audition-only condition showing the largest overshooting. In contrast, the younger group did not show significant overshooting in any condition.

### Variability

**FIGURE 6:**
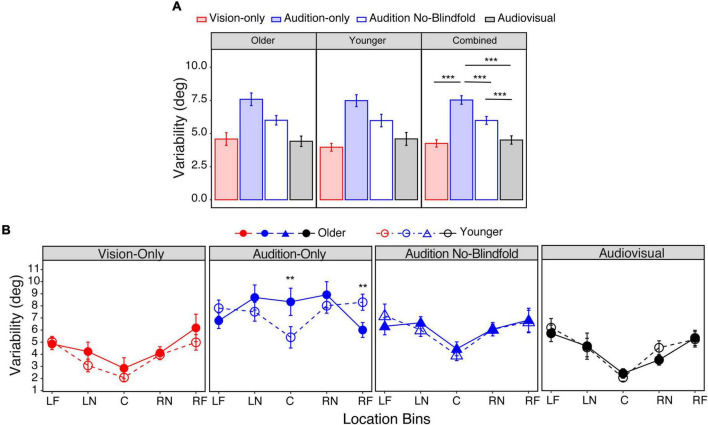
Variability. **(A)** The average variability across all seventeen locations in the four conditions for the older group (left panel), younger group (middle panel), and both groups combined (right panel). **(B)** The average variabilities across the five bins in the four conditions for the two groups, respectively. The five bins correspond to –90° to –60° (LF), –50° to –20° (LN), –10° to 10° (C), 20°–50° (RN), 60°–90° (RF). The error bars represent standard errors. In **(A)** statistical asterisks represent significant difference between testing conditions. In **(B)** statistical asterisks represent significant difference between the two groups. ***p < 0.001; **p < 0.05.

Again, we first obtained the mean variability across all seventeen locations, with smaller values corresponding to higher precision. For the older group, the mean variability ranged from 4.4° in the audiovisual condition to 7.6° in the audition-only condition (Figure 6A, left panel). For the younger group, the mean variability ranged from 4.0° in the vision-only condition to 7.5° in the audition-only condition (Figure 6A, middle panel). LME modeling showed a significant main effect of condition [*F*(3,78) = 42.0, *p* < 0.001], but not a main effect of group [*F*(1,26) = 0.27, *p* = 0.61] nor an interaction between condition and group [*F*(3,78) = 0.48, *p* = 0.69]. *Post hoc* analyses showed that the audition-only condition had significantly larger variability than the vision-only and audition no-blindfold conditions (*ps* < 0.001, Figure 6A, right panel), and that the audiovisual condition had similar variability as the vision-only condition (*p* = 1.00). Consistent with the absolute error analyses, these results suggested (1) an overall similar variability between the younger and older groups; (2) smaller visual than auditory localization variability; (3) a beneficial role of visual context for auditory localization, and (4) little to no benefit of auditory input when localizing audiovisual targets.

However, age differences appeared when considering localization variability across the target locations. To increase the statistical power, we grouped the seventeen locations into five bins corresponding to left far periphery (LF: −90° to −60°), left near periphery (LN: −50° to −20°), center (C: −10° to 10°), right near periphery (RN: 20°–50°), and right far periphery (RF: 60°–90°). As shown in Figure 6B, in the vision-only, audiovisual, and audition no-blindfold conditions, the variability increased from central to peripheral bins for both groups. However, in the audition-only condition, the older group’s variability averaged 8.3° in the central bin and reduced to 6.0° in the far peripheral bins; while the younger group’s variability averaged 5.4° in the central bin and increased to 8.3° in the far peripheral bins (Figure 6B). LME modeling showed significant main effects of location [*F*(4,494) = 16.10, *p* < 0.001] and condition [*F*(3,494) = 58.86, *p* < 0.001], an interaction between location and condition [*F*(12,494) = 2.38, *p* = 0.005], and a significant interaction between location and group [*F*(4,494) = 2.43, *p* = 0.047]. *Post hoc* analyses confirmed that in the audition-only condition, the older group had significantly larger variability than the younger group in the central bin (*p* = 0.003), but significantly smaller variability in the right far peripheral bin (*p* = 0.022). Figure 6B also shows group differences when comparing vision-only and audition-only conditions across the bins, therefore we conducted *post hoc* analyses to examine this effect. The younger group showed significantly smaller variability in the vision-only condition than the audition-only condition throughout the five bins (*ps* < 0.05), however, the older group only showed this vision advantage in the central and near peripheral bins (*ps* < 0.001).

### Audiovisual Integration

To further examine whether the localization of audiovisual targets was optimal, we calculated the optimal variability in the audiovisual condition per subject based on the Maximum-Likelihood Estimation (MLE) model (Battaglia et al., 2003; Rohde et al., 2016). Specifically, the MLE model predicts that an optimal observer will integrate audiovisual stimuli by assigning more weight to the modality with smaller variability (higher precision) and show smaller audiovisual variability than either individual modality, specifically proportional to the product of the unimodal variances divided by their sum (Eq. 1).


(1) $$\sigma_{AV}^{2}=\frac{\sigma_{A}^{2}\sigma_{V}^{2}}{\sigma_{A}^{2}+\sigma_{V}^{2}}$$

**FIGURE 7:**
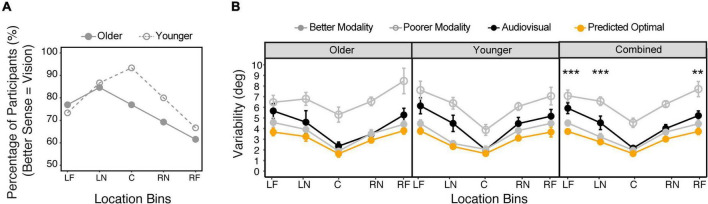
Audiovisual integration. **(A)** The percentage of subjects who showed vision as the better modality (smaller variability) at each location bin in each group. **(B)** Comparison between the actual audiovisual variability, predicted optimal variability based on the MLE model, variability of the better modality, and variability of the poorer modality, for the older and younger groups, respectively. The five bins correspond to –90° to –60° (LF), –50° to –20° (LN), –10° to 10° (C), 20°–50° (RN), 60°–90° (RF). The error bars represent standard errors. Statistical asterisks represent significant difference between the actual audiovisual variability and the predicted optimal variability. ***p < 0.001; **p < 0.05.

Note that we used the auditory no-blindfold condition to calculate standard deviations for this analysis. We compared the vision-only and auditory no-blindfold condition to determine the better and poorer single modalities for each individual subject at each location bin. Figure 7A shows the percentage of subjects whose vision was the better of the two senses at each bin. For the younger group, almost all subjects were vision dominant at the central bin (93%), while at peripheral bins the percentage of vision dominance dropped to approximately 70%. The older group overall had weaker vision dominance than the younger group, with the highest percentage of 85% at LN bin and lowest percentage of 62% at RF bin.

Figure 7B shows the comparison among the actual audiovisual variability, the predicted optimal variability, and the better and poorer single modality variabilities determined individually for each bin. LME modeling on the variability showed significant main effect of condition [*F*(3,36) = 64.0, *p* < 0.001], location [*F*(4,29) = 14.7, *p* < 0.001], and a significant interaction between condition and bins [*F*(12,352) = 1.85, *p* = 0.040]. There was no main effect of group or its interaction with other factors. *Post hoc* analyses were thus conducted on the combined data (Figure 7B). The results showed that the predicted optimal variability was always close to the better sense (*ps* > 0.05 in all bins), indicating that an optimal strategy was to ignore the less dominant sense (winner-takes-all, Battaglia et al., 2003). However, the actual audiovisual variability was only consistent with the optimal variability at the central and RN bins (*ps* > 0.05), but was significantly larger at the RF, LN, and LF bins (*ps* < 0.05). Moreover, at the LN and LF bins the audiovisual variability was larger than the better single modality (*ps* < 0.05), indicating a deviation from a “winner-take-all” strategy.

## Discussion

In this study, we used a spatial localization task to investigate the impact of typical aging on the egocentric localization of visual and auditory targets, and on the relative reliabilities of, and integration strategies for, the two senses. Although older and younger subjects had similar overall localization accuracy and variability within each tested modality, their performance differed at different stimulus locations, perhaps reflecting differences in selective attention, response strategies, or other cognitive processes.

### Age Effects on Unimodal Localization

Unlike younger subjects, older subjects showed more frequent misses of visual targets in the far peripheral field compared to the central field. It has been shown that older subjects show light sensitivity decline in peripheral vision despite normal visual acuity (Grobbel et al., 2016). However, we used large and high contrast visual stimuli, and none of the older subjects missed all four trials at any peripheral location, indicating that the higher miss rate was unlikely due to peripheral loss of light sensitivity. Instead, the higher miss rate at peripheral locations is consistent with previously reported shrinkage of the useful field of view, possibly due to visual attention deficits in peripheral fields in older individuals (Sekuler and Ball, 1986; Sekuler et al., 2000). However, we are the first to report this attention deficit is possibly specific to visual modality.

A significant age effect was also shown in auditory localization without vision. The older group showed significantly higher variability (less precise localization) than the younger group at central locations. Although our older subjects had age-adjusted normal hearing thresholds, they did show higher hearing thresholds than younger subjects especially at high frequencies. It is possible that they had reduced access to interaural level difference cues due to the high frequency hearing loss (Middlebrooks and Green, 1991). To further explore this possibility, we conducted a correlation analysis between the variability at the central bin and hearing threshold across high frequencies (4 k–8 kHz). There was no significant correlation (*r* = 0.30, *p* = 0.15), indicating that the reduced high frequency sensitivity may not explain the impaired sound localization abilities. This is consistent with previous studies that reported that hearing thresholds were poor predictors for sound localization performance (Noble et al., 1997; Akeroyd and Whitmer, 2016). More surprisingly, our older group had less variability (more precise localization) than the younger group at the right far peripheral location, further indicating that sound localization performance cannot be predicted solely based on the pure-tone thresholds.

These age effects on sound localization were unlikely to be induced by the specific parameters of the auditory stimuli we used. Our auditory stimuli were pink noise (200–8 kHz) at 60 dB SPL. Pink noise represents the energy distribution of human hearing, and is also representative of the broadband sounds in daily environments. Moreover, localizing pink noise stimuli can rely on all known spatial localization cues: interaural time difference, interaural intensity difference, and monaural spectral cues. It is not likely that inclusion of higher frequencies (>8 kHz) will cause larger deficits in horizontal localization in older subjects. In a previous study, Dobreva et al. (2011) showed that both younger and older subjects had similar performance when localizing broadband noise (100–20 kHz) and limited broadband noise similar to ours (100–10 kHz). The sound level (60 dB SPL) was suprathreshold for all subjects in this study. Previous literature showed that localization of broadband noise was generally not affected by sound intensity, except near threshold (Macpherson and Middlebrooks, 2000; Inoue, 2001; Sabin et al., 2005). However, when testing subjects with hearing disorders the sound should be adjusted to ensure audibility.

Older adults also showed increased overshooting in central space (i.e., they reported stimuli presented near central space as more peripheral than they were) regardless of target modality. Such overshooting has been previously reported in auditory localization and has also been shown to be larger in older subjects than younger subjects (Razavi et al., 2007; Cui et al., 2010; Dobreva et al., 2011). Dobreva et al. (2011) explained the overshooting in the auditory localization as a result of gaze-guided action when executing a pointing response. However, our study used verbal report instead of hand pointing, and the subjects were instructed to face forward throughout each trial. Moreover, the overshooting was most prominent in the auditory condition when the subjects were blindfolded, in which case there was no visual guidance at all. Therefore, our results indicate that overestimating the eccentricity of central stimuli may be a more general age-related change in localization performance. This effect is unlikely due to a response strategy or different understanding of verbally labeled directions, which would then predict similar overestimation across modalities. Future research should explore whether this effect is perceptual or strategic.

### Age Effects on the Interactions Between Vision and Hearing in Spatial Localization

Although the younger group showed consistently smaller variability in vision than audition regardless of the target location, the older group had smaller visual than auditory variability only at central and near peripheral locations, with comparable variability for visual and auditory stimuli at far peripheral locations. These findings suggest the presence of age-related changes in the advantage of vision over audition in spatial localization.

Testing auditory localization both with and without a blindfold allowed us to discriminate between two hypotheses about the role of visual context in auditory localization: first, that people might be better at concentrating on the sound with no access to visual distractions while wearing the blindfold, therefore performing better; second, that the visual context might provide an external reference for sound localization, in which case people would be better without the blindfold. Our results supported the latter hypothesis. Older and younger subjects both had increased accuracy (by 1.8 and 1.4°, respectively) and reduced variability (by 1.8 and 1.5°, respectively) when localizing sound without a blindfold. A previous study reported improved performance in an auditory localization task after preview of the test room and suggested that the room preview provided information about acoustic cues such as reverberations, which improved sound localization performance (Tonelli et al., 2015). However, our study was conducted in a sound-attenuated chamber where the reverberation was minimized. Another study reported improved hand and head pointing to sound locations without blindfolds by younger subjects in a sound-attenuated chamber, and explained the benefit as a result of visually guided motion during full viewing (Tabry et al., 2013). However, our subjects responded verbally rather than by pointing, meaning the benefit of visual context is not limited to guiding motor responses. Rather, our results suggest the visual context might play a fundamental reference for spatial localization instead of or in addition to providing environmental acoustic cues and guiding motor actions. In our study, there was no visual fixation point that could assist with the head or eye alignment in the no-blindfold condition, but subjects might have used the visible room locations or even the boundary of their visual field as references to anchor their auditory responses.

We also examined evidence for optimal sensory integration in our subjects. Unlike most past investigations, we did not intentionally degrade stimuli to increase localization uncertainty, instead focusing on localization uncertainty inherent in stimuli that were relatively easy to localize (Parise et al., 2012). Moreover, we examined the sensory integration across the entire frontal horizontal plane, instead of a restricted central space. We found that MLE modeling predicted as optimal a “winner-take-all” strategy, due to the large differences in variabilities between the two single modalities. We replicated results consistent with an optimal winner-take-all strategy in the central space for both groups, while providing evidence for sub-optimal integration in peripheral space. Interestingly, in peripheral space we found that both groups’ audiovisual performance was less precise than the better single modality. Previous literature has attributed suboptimal integration to misinterpretation of causal inference or different response strategies (e.g., different cost function to account for the speed-accuracy trade-off) (Meijer et al., 2019; for a topical discussion, see Rahnev and Denison, 2018). However, in our study we explicitly informed the subjects that the visual and auditory stimuli would appear at the same locations in the bimodal condition, and there was no requirement for reaction time or feedback for accuracy. It is more plausible that our subjects had erroneous weight assignments to the visual and auditory inputs at peripheral locations. As shown in Figure 7A, the better sense with smaller variability is location dependent. It is possible that the subjects assigned weights to vision and audition solely based on the relative reliabilities in central location (e.g., more weights to vision), even though peripheral locations increasingly favor audition. In all cases, these strategies did not differ significantly between older and younger adults.

### Implications for Testing Older Subjects With Vision and/or Hearing Disorders

An important goal of this study was to examine the feasibility of the current research method for investigating the impact of vision and/or hearing impairment on spatial localization. Due to our interest in people with sensory impairment, there are several important differences between our testing paradigm and others. Our subjects responded verbally, rather than pointing or walking to locations as has been used in several past studies (e.g., Dobreva et al., 2011; Tabry et al., 2013). Verbal report is less mobility-demanding than pointing and walking, making it easier for many subjects who are older, have sensory impairment, or who have mobility constraints. In addition, the actions during pointing/walking may introduce additional errors or biases related to motor systems rather than perception (Soechting and Flanders, 1992). Verbal report also better probes the on-line perception of egocentric direction, while visually guided motion (e.g., pointing or walking) has greater demands on memory because subjects have to map their motion to the remembered spatial location (Fortenbaugh et al., 2008; Philbeck et al., 2008).

Our subjects were asked to face straight-ahead prior to the start of each trial to ensure that the stimuli were presented at the intended location in relation to the subjects. However, we did not control the subjects’ head and eye movements due to the following considerations. In pilot testing, we found that fixing head movements using a chin or forehead rest may be impractical and cause unacceptable fatigue in some older subjects. More importantly, many of our subjects with vision impairment in the next stage of this study may have visual field loss, such as central field loss due to age-related macular degeneration. People with central visual field loss may have different habitual facing and gazing directions when asked to “face straight-ahead.” It would be interesting to examine whether such strategy would cause systematic biases in their sound localization, as head orientation affects interaural differences, and previous studies have shown that gaze directions in subjects with normal vision can also affect their sound localization (Razavi et al., 2007; Dobreva et al., 2012). One limitation of not fixing subjects’ head and eye movements is that our subjects may have turned their head and eyes after the onset of stimulus presentation. However, this is not likely for our short stimulus presentation time (200 ms), which was not sufficient for saccades or head movements (Becker and Jürgens, 1979). But we acknowledge that preparatory eye or head motor signals, if any, cannot be entirely ruled out and may have affected the subjects’ localization performance. Nevertheless, the highly similar results across vision-only, auditory-no blindfold, and audiovisual conditions indicate that our subjects had consistent head and gaze control under these conditions.

All younger and older subjects in this study self-reported having normal vision and hearing, and their acuity, contrast sensitivity, and hearing threshold were confirmed to be within the normal age-adjusted range. However, we acknowledge that it is difficult to draw an absolute boundary between normal aging and age-related sensory impairment. For example, older individuals often have reduced light sensitivity especially at peripheral vision despite “normal vision” based on standard criteria (Grobbel et al., 2016), and increased hearing thresholds at high sound frequencies despite “normal hearing” based on standard criteria (International Organization for Standardization [ISO], 2000). When testing older subjects with vision and/or hearing disorder, higher visual miss rates in the periphery in our results may be confounded with subjects’ peripheral field loss, and the impaired sound localization performance may be confounded with the subjects’ elevated hearing threshold. To address these issues, the saliency of the visual and auditory stimuli may need to be adjusted in future studies to ensure visibility and audibility for subjects with vision and/or hearing disorders. Precise visual field test may be required to screen subjects with subtle ocular pathology that affects peripheral field (e.g., early stage glaucoma). Besides the sensory decline, older individuals often show other functional decline such as impaired spatial attention and temporal processing, which can also affect their spatial localization performance. These confounding factors highlight the necessity to build a baseline of typical aging for older individuals with vision and/or hearing impairment.

## Conclusion

This study provided an exploration of the role of typical aging in spatial localization of visual, auditory, and audiovisual stimuli. In younger and older adults without sensory loss, mean localization performance as measured by accuracy and variability was similar across age groups, for both visual and auditory localization. However, older and younger adults did not show the same pattern of errors in spatial localization as a function of stimulus location, suggesting that average metrics that do not take into account localization at different locations in space may not adequately reflect age-related changes in spatial perception. Specifically, older adults tended to localize central stimuli as more peripheral than they actually were, a pattern that was consistent across modalities and absent in younger adults. Moreover, older adults showed larger variability at central locations and smaller variability at peripheral locations when localizing sound without vision, a pattern that deviated from younger adults. Older adults also missed peripheral visual stimuli at significantly higher rates than younger adults, reflecting a potentially modality-specific deficit in visual attention to the periphery due to aging. Future research should aim to uncover the mechanisms and effects of each of these differences using more targeted paradigms. In addition, we believe that the success of this paradigm in uncovering age differences in spatial localization serves as validation that similar approaches may be useful for understanding localization differences in people with sensory loss.

## Data Availability Statement

The raw data supporting the conclusions of this article will be made available by the authors, without undue reservation.

## Ethics Statement

The studies involving human participants were reviewed and approved by the University of Minnesota Institutional Review Board. The patients/participants provided their written informed consent to participate in this study.

## Author Contributions

Y-ZX, DA, PBN, and GL designed the research. Y-ZX, DA, and NN performed the research. Y-ZX and DA analyzed the data. Y-ZX, DA, and GL wrote and revised the manuscript. PBN and NN provided editorial suggestions. All authors approved the final version of the manuscript.

## Conflict of Interest

The authors declare that the research was conducted in the absence of any commercial or financial relationships that could be construed as a potential conflict of interest.

## Publisher’s Note

All claims expressed in this article are solely those of the authors and do not necessarily represent those of their affiliated organizations, or those of the publisher, the editors and the reviewers. Any product that may be evaluated in this article, or claim that may be made by its manufacturer, is not guaranteed or endorsed by the publisher.

## References

[B1] Akeroyd M. A. (2014). An overview of the major phenomena of the localization of sound sources by normal-hearing, hearing-impaired, and aided listeners. *Trends Hear.* 18 10–16. 10.1177/2331216514560442 25492094 PMC4271773

[B2] Akeroyd M. A. Whitmer W. M. (2016). “Spatial hearing and hearing aids,” in *Hearing Aids*, eds Popelka G. R. Moore B. C. J. Fay R. R. Popper A. N. (Cham: Springer International Publishing), 181–215. 10.1007/978-3-319-33036-5_7

[B3] Andersen G. J. (2012). Aging and vision: changes in function and performance from optics to perception. *Wiley Interdiscip. Rev. Cogn. Sci.* 3 403–410. 10.1002/wcs.1167 22919436 PMC3424001

[B4] Andres P. Parmentier F. B. Escera C. (2006). The effect of age on involuntary capture of attention by irrelevant sounds: A test of the frontal hypothesis of aging. *Neuropsychologia* 44 2564–2568. 10.1016/j.neuropsychologia.2006.05.005 16797613

[B5] Battaglia P. W. Jacobs R. A. Aslin R. N. (2003). Bayesian integration of visual and auditory signals for spatial localization. *J. Optical Soc. Am. A* 20 1391–1397. 10.1364/josaa.20.001391 12868643

[B6] Baum S. H. Stevenson R. (2017). Shifts in Audiovisual Processing in Healthy Aging. *Curr. Behav. Neurosci. Rep.* 4 198–208. 10.1007/s40473-017-0124-7 29862161 PMC5972362

[B7] Becker W. Jürgens R. (1979). An analysis of the saccadic system by means of double step stimuli. *Vis. Res.* 199 1967–1983. 10.1016/0042-6989(79)90222-0 532123

[B8] Bennett C. R. Loomis J. M. Klatzky R. L. Giudice N. A. (2017). Spatial updating of multiple targets: comparison of young and older adults. *Memory Cogn.* 45 1240–1251. 10.3758/s13421-017-0725-0 28653274 PMC5711592

[B9] Cienkowski K. M. Carney A. E. (2002). Auditory–visual speech perception and aging. *Ear Hear.* 23 439–449. 10.1097/00003446-200210000-00006 12411777

[B10] Cui Q. N. Razavi B. O’Neill W. E. Paige G. D. (2010). Perception of auditory, visual, and egocentric spatial alignment adapts differently to changes in eye position. *J. Neurophysiol.* 103 1020–1035. 10.1152/jn.00500.2009 19846626 PMC2822679

[B11] Diederich A. Colonius H. Schomburg A. (2008). Assessing age-related multisensory enhancement with the time-window-of-integration model. *Neuropsychologia* 46 2556–2562. 10.1016/j.neuropsychologia.2008.03.026 18490033

[B12] Dobreva M. O’Neill W. E. Paige G. D. (2011). Influence of aging on human sound localization. *J. Neurophysiol.* 105 2471–2486. 10.1152/jn.00951.2010 21368004 PMC3094163

[B13] Dobreva M. S. O’Neill W. E. Paige G. D. (2012). Influence of age, spatial memory, and ocular fixation on localization of auditory, visual, and bimodal targets by human subjects. *Exp. Brain Res.* 223 441–455. 10.1007/s00221-012-3270-x 23076429

[B14] Ernst M. O. Banks M. S. (2002). Humans integrate visual and haptic information in a statistically optimal fashion. *Nature* 415 429–433. 10.1038/415429a 11807554

[B15] Ferris F. L. III Kassoff A. Bresnick G. H. Bailey I. (1982). New visual acuity charts for clinical research. *Am. J. Ophthalmol.* 94 91–96. 10.1016/0002-9394(82)90197-0 7091289

[B16] Fortenbaugh F. C. Hicks J. C. Turano K. A. (2008). The effect of peripheral visual field loss on representations of space: evidence for distortion and adaptation. *Invest. Ophthalmol. Vis. Sci.* 49 2765–2772. 10.1167/iovs.07-1021 18515599

[B17] Freigang C. Richter N. Rübsamen R. Ludwig A. A. (2015). Age-related changes in sound localisation ability. *Cell Tissue Res.* 361 371–386. 10.1007/s00441-015-2230-8 26077928

[B18] Freigang C. Schmiedchen K. Nitsche I. Rubsamen R. (2014). Free-field study on auditory localization and discrimination performance in older adults. *Exp. Brain Res.* 232 1157–1172. 10.1007/s00221-014-3825-0 24449009

[B19] Fuller S. D. Mudie L. I Siordia C. Swenor B. K. Friedman D. S. (2018). Nationwide prevalence of self-reported serious sensory impairments and their associations with self-reported cognitive and functional difficulties. *Ophthalmology* 125 476–485. 10.1016/j.ophtha.2017.11.003 29306552

[B20] Gallun F. J. (2021). Impaired Binaural Hearing in Adults: A Selected Review of the Literature. *Front. Neurosci.* 19:610957. 10.3389/fnins.2021.610957 33815037 PMC8017161

[B21] Grobbel J. Dietzsch J. Johnson C. A. Vonthein R. Stingl K. Weleber R. G. (2016). Normal values for the full visual field, corrected for age and reaction time, using semiautomated kinetic testing on the octopus 900 perimeter. *Transl. Vis. Sci. Technol.* 5:5. 10.1167/tvst.5.2.5 26966641 PMC4782826

[B22] Inoue J. (2001). Effects of stimulus intensity on sound localization in the horizontal and upper-hemispheric median plane. *J. UOEH.* 23 127–138. 10.7888/juoeh.23.127 11431958

[B23] International Organization for Standardization [ISO] (2000). *Acoustics: Statistical Distribution of Hearing Thresholds as a Function of Age. ISO 7029.* Geneva: International Organization for Standardization.

[B24] Jones S. A. Noppeney U. (2021). Ageing and multisensory integration: A review of the evidence, and a computational perspective. *Cortex* 138 1–23. 10.1016/j.cortex.2021.02.001 33676086

[B25] Kersten D. Yuille A. L. (2003). Bayesian models of object perception. *Curr. Opin. Neurobiol.* 13 50–158. 10.1016/s0959-4388(03)00042-4 12744967

[B26] King A. J. (2009). Visual influences on auditory spatial learning. *Phil. Trans. R. Soc. B* 364 331–339. 10.1098/rstb.2008.0230 18986967 PMC2674475

[B27] Long R. G. Giudice N. A. (2010). “Establishing and maintaining orientation for mobility,” in *Foundations of Orientation and Mobility*, 3rd Edn, eds Blasch B. B. Wiener W. R. Welsh R. W. (New York: American Foundation for the Blind), 45–62.

[B28] Loomis J. M. Klatzky R. L. McHugh B. Giudice N. A. (2012). Spatial working memory for locations specified by vision and audition: testing the amodality hypothesis. *Atten. Percept. Psychophys.* 74 1260–1267. 10.3758/s13414-012-0311-2 22552825 PMC3482114

[B29] Macpherson E. A. Middlebrooks J. C. (2000). Localization of brief sounds: effects of level and background noise. *J. Acoust. Soc. Am.* 108 1834–1849. 10.1121/1.1310196 11051510

[B30] Meijer D. Veselič S. Calafiore C. Noppeney U. (2019). Integration of audiovisual spatial signals is not consistent with maximum likelihood estimation. *Cortex* 119 74–88. 10.1016/j.cortex.2019.03.026 31082680 PMC6864592

[B31] Meng Q. Wang B. Cui D. Liu N. Huang Y. Chen L. (2019). Age-related changes in local and global visual perception. *J. Vis.* 19:10. 10.1167/19.1.10 30650433

[B32] Middlebrooks J. C. Green D. M. (1991). Sound localization by human listeners. *Annu. Rev. Psychol.* 42 135–159. 10.1146/annurev.ps.42.020191.001031 2018391

[B33] Murray M. M. Eardley A. F. Edginton T. Oyekan B. Smyth E. Matusz P. J. (2018). Sensory dominance and multisensory integration as screening tools in aging. *Sci. Rep.* 8:8901. 10.1038/s41598-018-27288-2 29891964 PMC5995929

[B34] Murray M. M. Wallace M. (2011). “Multisensory integration and aging,” in *The Neural Bases of Multisensory Processes*, eds Micah M. M. Mark T. W.. (Boca Raton: CRS Press).

[B35] Noble W. Byrne D. Ter-Horst K. (1997). Auditory localization, detection of spatial separateness, and speech hearing in noise by hearing impaired listeners. *J. Acoust. Soc. Am*. 102 2343–2352. 10.1121/1.419618 9348693

[B36] Parise C. V. Spence C. Ernst M. O. (2012). When correlation implies causation in multisensory integration. *Curr. Biol.* 22 46–49. 10.1016/j.cub.2011.11.039 22177899

[B37] Pelli D. G. (1997). The VideoToolbox software for visual psychophysics: transforming numbers into movies. *Spat. Vis.* 10 437–442. 10.1163/156856897x00366 9176953

[B38] Pelli D. G. Robson J. G. Wilkins A. J. (1988). The design of a new letter chart for measuring contrast sensitivity. *Clin. Vis. Sci.* 2 187–199.

[B39] Philbeck J. Sargent J. Arthur J. Dopkins S. (2008). Large manual pointing errors, but accurate verbal reports, for indications of target azimuth. *Perception* 37 511–534. 10.1068/p5839 18546661 PMC2702262

[B40] Piepho H. P. (2004). An algorithm for a letter-based representation of all pairwise comparisons. *J. Comput. Graph. Stat.* 13 456–466. 10.1198/1061860043515 12611515

[B41] Pinheiro J. Bates D. (2000). *Mixed-Effects Models in S and S-PLUS.* Berlin: Springer.

[B42] Postma A. Van Der Ham I. J. M. (2017). *Neuropsychology of Space: Spatial Functions of the Human Brain.* San Diego: Academic Press.

[B43] R Core Team (2005). *R: A Language and Environment for Statistical Computing.* Vienna: R Foundation for Statistical Computing.

[B44] Rahnev D. Denison R. N. (2018). Suboptimality in perceptual decision making. *Behav. Brain Sci.* 41:e223. 10.1017/S0140525X18000936 PMC6110994 29485020

[B45] Razavi B. O’Neill W. E. Paige G. D. (2007). Auditory spatial perception dynamically realigns with changing eye position. *J. Neurosci.* 27 249–258. 10.1523/JNEUROSCI.0938-07.2007 17881531 PMC6672669

[B46] Recanzone G. (2018). The effects of aging on auditory cortical function. *Hear. Res.* 366 99–105. 10.1016/j.heares.2018.05.013 29853323 PMC6103827

[B47] Rohde M. Van Dam L. C. J. Ernst M. O. (2016). Statistically Optimal Multisensory Cue Integration: A Practical Tutorial. *Multisens. Res.* 29 279–317. 10.1163/22134808-00002510 29384605

[B48] Sabin A. T. Macpherson E. A. Middlebrooks J. C. (2005). Human sound localization at near-threshold levels. *Hear. Res.* 199 124–134. 10.1016/j.heares.2004.08.001 15574307

[B49] Saunders G. H. Echt K. V. (2007). An overview of dual sensory impairment in older adults: perspectives for rehabilitation. *Trends Amplif.* 11 243–259. 10.1177/1084713807308365 18003868 PMC4111537

[B50] Sekuler A. B. Bennett P. J. Mamelak M. (2000). Effects of aging on the useful field of view. *Exp. Aging Res.* 26 103–120. 10.1080/036107300243588 10755218

[B51] Sekuler R. Ball K. (1986). Visual localization: age and practice. *J. Optical Soc. Am. A* 3 864–867. 10.1364/josaa.3.000864 3734925

[B52] Simon H. J. Levitt H. (2007). Effect of dual sensory loss on auditory localization: implications for intervention. *Trends Amplif.* 11 259–272. 10.1177/1084713807308209 18003869 PMC4111533

[B53] Soechting J. F. Flanders M. (1992). Moving in three-dimensional space: frames of references, vectors, and coordinate systems. *Annu. Rev. Neurosci.* 15 167–191. 10.1146/annurev.ne.15.030192.001123 1575441

[B54] Subramanian A. Dickinson C. (2006). Spatial localization in visual impairment. *Invest. Ophthalmol. Vis. Sci.* 47 78–85. 10.1167/iovs.05-0137 16384947

[B55] Tabry V. Zatorre R. J. Voss P. (2013). The influence of vision on sound localization abilities in both the horizontal and vertical planes. *Front. Psychol.* 4:932. 10.3389/fpsyg.2013.00932 24376430 PMC3860057

[B56] Tonelli A. Brayda L. Gori M. (2015). Task-dependent calibration of auditory spatial perception through environment visual observation. *Front. Syst. Neurosci.* 9:84. 10.3389/fnsys.2015.00084 26082692 PMC4451354

[B57] Voss P. (2016). Auditory spatial perception without vision. *Front. Psychol.* 7:1960. 10.3389/fpsyg.2016.01960 28066286 PMC5167702

